# Microsurgical management of anterior clinoidal meningioma with internal carotid artery compression following pregnancy-associated tumor growth

**DOI:** 10.3171/2025.10.FOCVID25130

**Published:** 2026-01-01

**Authors:** Toshikazu Kimura, Shunsuke Ichi

**Affiliations:** Department of Neurosurgery, Japanese Red Cross Medical Center, Shibuya, Tokyo, Japan

**Keywords:** anterior clinoidal meningioma, pregnancy, visual disturbance, pregnancy-associated tumor growth, internal carotid artery stenosis, optic nerve compression

## Abstract

Meningiomas may enlarge during pregnancy. This video presents a case of an anterior clinoidal meningioma that showed rapid growth during a patient’s second trimester of pregnancy, leading to marked stenosis of the internal carotid artery (ICA). In clinoidal meningiomas, early identification of the proximal ICA and middle cerebral artery is essential for safe orientation and efficient resection. However, a narrowed ICA increases the risk of dissection and, in young patients with large tumors, securing distal control can be challenging. This video highlights technical nuances for safe dissection in such cases.

The video can be found here: https://stream.cadmore.media/r10.3171/2025.10.FOCVID25130

## Transcript

In this video, we present the case of a rapidly enlarging anterior clinoidal meningioma during pregnancy.^1^ The tumor caused progressive visual loss and compression of the internal carotid artery (ICA), ultimately requiring surgical resection shortly after delivery.

This is the case of a 29-year-old woman who was 17 weeks pregnant when she began experiencing some visual disturbance in her left eye. An MRI revealed a brain tumor, and she was referred to our hospital. We considered early resection, but after a thorough discussion with both the obstetric team and the patient, we decided that even if the tumor got a bit larger, the risk of losing vision or increasing surgical difficulty would likely stay low.^2^ So we prioritized the baby’s development and chose to continue the pregnancy while keeping a close eye on the tumor with imaging.

Three months later, the MRI showed that the tumor had grown significantly and her visual field had narrowed further. On MRA, the left middle cerebral artery (MCA) was no longer visible. We abandoned the goal of reaching full term and made a plan to deliver at 34 weeks. We also decided that if her neurological condition worsened before that, we would proceed with emergency surgery.

A cesarean section was performed on day 0 of week 34, and 5 days later, we proceeded with tumor resection. By that time, her left visual acuity had declined to light perception only, and she showed mild disorientation. The tumor had further enlarged, reaching a diameter of 8 cm.

### 1:50 Surgical Plan.

Our surgical strategy was structured in six key steps, as outlined here. This stepwise approach allowed for safe exposure and resection while preserving neurovascular structures.

### 2:00 Patient Prepared for Surgery.

The patient was placed in the supine position with the backboard elevated 20° and fixed with a 3-point head holder. We started with a left frontotemporal craniotomy. After identifying the superior orbital fissure, we drilled out the cancellous bone inside the anterior clinoid process. After dissecting from surrounding dura, the anterior clinoid process was removed.^3,4^

### 2:26 Opening the Optic Sheath.

Then, the optic canal was unroofed. A small incision was made in the frontal dura near the optic canal, followed by cutting of the falciform ligament and opening of the optic nerve sheath. Tumor tissue that had extended into the optic canal was removed to decompress the optic nerve.^5^

### 2:47 Decompression of the Tumor.

Next, we opened the dura and began internal decompression of the tumor along the skull base. The tumor was relatively soft, but not soft enough to be aspirated. It was also highly vascular and bled easily, so we had to repeatedly remove small portions and perform meticulous hemostasis each time. Using bipolar coagulation and other standard techniques, we proceeded with resection.

We attempted to secure the distal MCA, but significant brain swelling made this difficult. We continued decompression while controlling bleeding. In addition to bipolar coagulation, Surgicel and Floseal were used for temporary hemostasis. We estimated the thickness of the tumor and performed repeated, localized internal decompression to avoid injuring deeper vessels, searching the branch of the MCA.

As the tumor volume decreased, a space opened up between the frontal lobe and the tumor, allowing us to identify an M3 branch. Now that the MCA could be traced proximally, we could estimate the tumor thickness more precisely and proceeded with internal debulking. With continued decompression, we could visualize the MCA bifurcation and trace it proximally to identify the perforators.

### 4:24 Dissection From the Frontal Lobe.

Eventually, we were able to identify the ICA and debulked the tumor around the frontal lobe. We then carefully dissected the tumor off the olfactory nerve to create more working space. After debulking the tumor on the medial side of the ICA, we carefully dissected the tumor and were able to visualize the optic nerve. And we could see the left A1 and recurrent artery of Heubner.

4:58 Dissection of the Important Branches of the ICA. After that, we clearly visualized the ICA. It appeared whitish and degenerated, which made us concerned about a possible risk of dissection. So, we made sure to decompress around the lateral side of the ICA thoroughly. After sufficient decompression was achieved, we carefully separated the tumor from the anterior choroidal artery.

We identified the oculomotor nerve and proceeded to carefully dissect around the distal anterior choroidal artery. The tumor was dissected from the M1 segment. We could gradually peel the tumor away from the posterior communicating artery. The tumor extending from the medial temporal lobe was also removed.

### 5:54 Resection of the Temporal Dura.

The middle fossa dura is now visible. We then returned to the superior orbital fissure and dissected the dura propria of the middle fossa. Bleeding from the cavernous sinus was controlled using Floseal and Surgicel. We repeated the process of gently peeling off the dura while meticulously achieving hemostasis and eventually resected the thickened dura.^6^ After opening the porous oculomotorius, we removed the tumor in that region as well. At this point, we could visualize the posterior side of the ICA clearly. Finally, we incised the dura on the frontal lobe side toward the sphenoid planum and completed the resection.

This was the final surgical view.

### 6:58 Postoperative Course.

On the first day after surgery, the patient’s visual acuity had improved to counting fingers. Postoperative MRI showed a gross-total resection, and there were no abnormalities on diffusion-weighted imaging. Three months later, she reported that the vision in her left eye still seemed a little darker than the right, but her visual field had improved significantly.

This case highlights the rare but important scenario of pregnancy-associated meningioma enlargement and the surgical considerations when ICA stenosis and visual loss are present. Early multidisciplinary decision-making and careful microsurgical technique were essential in achieving a favorable outcome.

Thank you for watching.

## Acknowledgments

We used Google cloud, Text-to-Speech for voiceover.

## Disclosures

The authors report no conflict of interest concerning the materials or methods used in this study or the findings specified in this publication.

## Author Contributions

Primary surgeon: Kimura. Editing and drafting the video and abstract: Kimura. Critically revising the work: Kimura. Reviewed submitted version of the work: Kimura. Approved the final version of the work on behalf of both authors: Kimura. Supervision: Ichi.

## Supplemental Information

### Patient Informed Consent

The necessary patient informed consent was obtained in this study.
